# PPAR-gamma agonist pioglitazone recovers mitochondrial quality control in fibroblasts from *PITRM1*-deficient patients

**DOI:** 10.3389/fphar.2023.1220620

**Published:** 2023-07-26

**Authors:** Alessia Di Donfrancesco, Christian Berlingieri, Marta Giacomello, Chiara Frascarelli, Ana Paula Magalhaes Rebelo, Laurence A. Bindoff, Segel Reeval, Paul Renbaum, Filippo M. Santorelli, Giulia Massaro, Carlo Viscomi, Massimo Zeviani, Daniele Ghezzi, Emanuela Bottani, Dario Brunetti

**Affiliations:** ^1^ Unità di Genetica Medica e Neurogenetica, Fondazione IRCCS Istituto Neurologico Carlo Besta, Milan, Italy; ^2^ Department of Biology, University of Padova, Padova, Italy; ^3^ Department of Biomedical Sciences, University of Padova, Padova, Italy; ^4^ Department of Clinical Medicine, University of Bergen, Bergen, Norway; ^5^ Shaare Zedek Medical Center, The Hebrew University of Jerusalem, Jerusalem, Israel; ^6^ Molecular Medicine, IRCCS Fondazione Stella Maris, Calambrone, Italy; ^7^ UCL School of Pharmacy, University College London, London, United Kingdom; ^8^ Department of Neurosciences, University of Padova, Padova, Italy; ^9^ Department of Pathophysiology and Transplantation, University of Milan, Milan, Italy; ^10^ Department of Diagnostic and Public Health, Section of Pharmacology, University of Verona, Verona, Italy; ^11^ Department of Medical Biotechnology and Translational Medicine, University of Milan, Milan, Italy

**Keywords:** cerebellar ataxia, mitochondrial disease, proteostasis, pioglitazone, neurodegenaration

## Abstract

**Introduction:** Biallelic variants in *PITRM1* are associated with a slowly progressive syndrome characterized by intellectual disability, spinocerebellar ataxia, cognitive decline and psychosis. The pitrilysin metallopeptidase 1 (PITRM1) is a mitochondrial matrix enzyme, which digests diverse oligopeptides, including the mitochondrial targeting sequences (MTS) that are cleaved from proteins imported across the inner mitochondrial membrane by the mitochondrial processing peptidase (MPP). Mitochondrial peptidases also play a role in the maturation of Frataxin, the protein affected in Friedreich’s ataxia. Recent studies in yeast indicated that the mitochondrial matrix protease Ste23, which is a homologue of the human insulin-degrading enzyme (IDE), cooperates with Cym1 (homologue of *PITRM1*) to ensure the proper functioning of the preprotein processing machinery. In humans, IDE could be upregulated by Peroxisome Proliferator-Activated Receptor Gamma (PPARG) agonists.

**Methods:** We investigated preprotein processing, mitochondrial membrane potential and MTS degradation in control and patients’ fibroblasts, and we evaluated the pharmacological effect of the PPARG agonist Pioglitazone on mitochondrial proteostasis.

**Results:** We discovered that PITRM1 dysfunction results in the accumulation of MTS, leading to the disruption and dissipation of the mitochondrial membrane potential. This triggers a feedback inhibition of MPP activity, consequently impairing the processing and maturation of Frataxin. Furthermore, we found that the pharmacological stimulation of PPARG by Pioglitazone upregulates IDE and also PITRM1 protein levels restoring the presequence processing machinery and improving Frataxin maturation and mitochondrial function.

**Discussion:** Our findings provide mechanistic insights and suggest a potential pharmacological strategy for this rare neurodegenerative mitochondrial disease.

## Graphical Abstract

**Figure:**
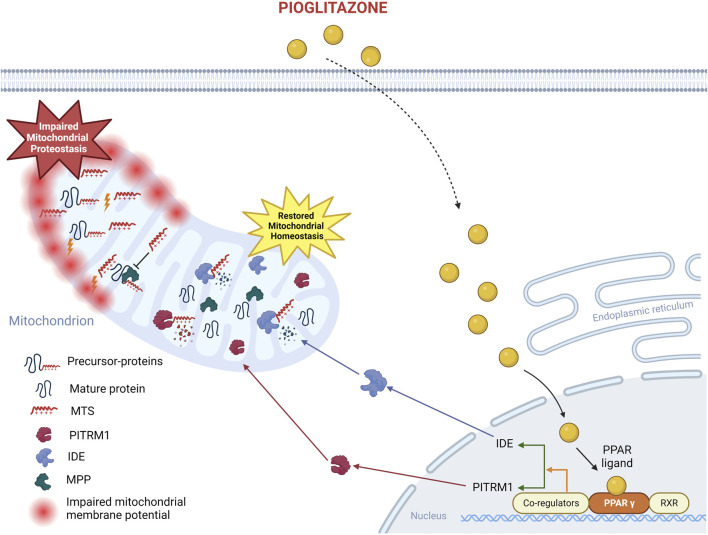
Proposed mechanism of action of pioglitazone on PITRM1^MUT^cells. The beneficial effects of pioglitazone have been confirmed in the current study on fibroblasts from PITRM1 patients: pioglitazone enhances PPARG protein level, which in turn upregulates IDE and PITRM1 restoring mitochondrial health. Created with BioRender.com.

## Introduction

Autosomal recessive cerebellar ataxias (ARCA) represent rare neurodegenerative conditions leading to imbalance and uncoordinated gait. The most common ARCA include ataxia-telangiectasia, ataxia oculomotor apraxia, and Friedreich’s ataxia. Ultrarare forms are characterized by a high genetic, biochemical, and clinical complexity, often characterized by early onset ataxia and intellectual disability (Anheim et al., 2010).

Currently, more than 107 genes are known to cause ARCA (Traschütz et al., 2023) however, despite recent progress in genetic diagnosis, about half of the patients remain undiagnosed. The combination of reduced diagnostic yield, with the high heterogeneity in genotype/phenotype correlations and a limited understanding of the pathophysiology hampers the opportunities for therapies in several forms (Cheng et al., 2021).

We have recently discovered that recessive *PITRM1* pathogenic variants are associated with a slowly progressive syndrome characterized by spinocerebellar ataxia, intellectual disability, impaired cognition and psychosis (Brunetti et al., 2016; Langer et al., 2018; Tolomeo et al., 2021).

*PITRM1* encodes a protein called pitrilysin metallopeptidase 1, also known as human presequence protease or hPreP. This protein, weighing 117 kDa, is located in the mitochondrial matrix and plays a crucial role in the breakdown of peptides up to 65 amino acids in length. It can degrade short unstructured peptides as well as various forms of amyloid-beta peptide (Aβ). Apart from its involvement in peptide degradation, PITRM1 functions as a scavenger, responsible for digesting the mitochondrial targeting sequence (MTS) of proteins that are imported across the inner mitochondrial membrane (Ståhl et al., 2002; Alikhani et al., 2011; Teixeira and Glaser, 2013), which are cleaved from the mature polypeptides by the mitochondrial matrix peptidase (MMP).

MTSs possess an amphiphilic nature characterized by a polar side that is positively charged and rich in arginine, and an opposite apolar side. This structural arrangement gives rise to a unique property of MTS peptides: when they accumulate within the mitochondrial matrix, they can exert a detergent-like effect and act as toxic agents. This is because they can form pores in the membranes, leading to the dissipation of the mitochondrial membrane potential (Brunetti et al., 2021). This mechanism was initially elucidated in a yeast model missing *CYM1*, the *PITRM1* homologue (Mossmann et al., 2014).

Deletion of the *CYM1* gene results in the impairment of MTS processing and the subsequent accumulation of MTS peptides. This accumulation triggers a feedback inhibition on the activity of MPP, which in turn leads to the buildup of immature precursor proteins. This accumulation of immature proteins hampers the maintenance of proper organellar proteostasis. In yeast, this defect was effectively rescued by overexpressing the mitochondrial isoform of Ste23, which is the equivalent of the human insulin-degrading enzyme (IDE). Ste23 is a mitochondrial matrix protease that plays a critical role in efficient peptide degradation (Taskin et al., 2017).

Recently, the knockout (KO) of *PITRM1* in human cells was reported to cause perturbed intermediated Frataxin processing (Kücükköse et al., 2021). Frataxin (FXN) is the protein missing in Friedreich’s ataxia (Clark et al., 2018). Frataxin processing involves two steps: the cytosolic precursor (pFXN) is initially cleaved by MPP to form an intermediate form (iFXN), and further processed to a mature protein (mFXN).

The mature form of Frataxin (mFXN) plays a crucial role in the formation of iron-sulfur (Fe-S) clusters, which serve as prosthetic groups in various mitochondrial and non-mitochondrial proteins. When the levels of mFXN are low, it results in decreased synthesis of Fe-S clusters. This, in turn, leads to the accumulation of iron within the mitochondria and compromises the antioxidant defense mechanisms. Ultimately, these processes contribute to the development of pathological conditions in the affected tissues.

We hypothesized that impaired Frataxin processing is also present in *PITRM1-*defective patients, and may play an important role in the pathogenesis of the ataxic phenotype. Therefore, we used fibroblasts obtained from *PITRM1-*mutated patients to study Frataxin processing and mitochondrial dysfunction.

Because a previous report showed that the human IDE promoter is a direct target of the Peroxisome proliferator-activated receptor gamma (PPARG) (Du et al., 2009), we tested the hypothesis that pharmacological stimulation of IDE by Pioglitazone, a PPARG agonist, can partially restore the presequence processing machinery and improve mitochondrial function.

## 2 Materials and methods

### 2.1 Ethics statement

This study is compliant with the guidelines of the Declaration of Helsinki and under protocols approved by the local ethics committees. Informed consent to use these samples for research was obtained in an anonymized way. PITRM1^R183Q^ cell line (here defined PT1) carrying homozygous missense mutations (c.548G>A, p.Arg183Gln) was provided by the University of Bergen (Norway) and previously described by us (Brunetti et al., 2016); PITRM1^V747Gfs*31^ cell line (here defined PT2) carrying homozygous c.2239dupG variant in *PITRM1* (p.Val747Glyfs*31) was provided by the IRCCS Fondazione Stella Maris (Pisa, Italy) and previously described by us (Tolomeo et al., 2021). PITRM1^T931M^ cell line (here defined PT3) carrying homozygous *PITRM1* mutation (c.2795C>T, p.Thr931Met) was provided by the Sharee Zedek Medical Center (Jerusalem, Israel) and previously described by us (Langer et al., 2018). See Table 1 for genotype-phenotype details. Controls cells were obtained by healthy donors at the Fondazione IRCCS Istituto Neurologico Carlo Besta (Milan, Italy).

**TABLE 1:** Summary of clinical findings in patients harbouring biallelic mutations in *PITRM1*(NM_0148989).

	PT 1	PT 2	PT 3
***PITRM1* variant**	c.548G>A; p.R183Q	c. 2239dupG; p.V747Gfs*31	c.2795C>T; p.T931M
**Ethnicity**	Norwegian	Italian	Palestinian Arab
**Gender**	Female	Male	Male
**Onset of disease**	Childhood	Infancy	Early childhood
**Symptoms at onset**	Mild intellectual disability	Seizures, developmental delay, hypotonia	Developmental delay, tremor
**Clinical findings**	Ataxic syndrome, cognitive impairment, psychosis	Ataxic syndrome, Intellectual disability	Ataxic syndrome, Intellectual disability
**Effect on PITRM1 expression**	Protein reduction	Protein reduction	Protein reduction

### 2.2 Cell culture and treatments

Skin fibroblasts from healthy subjects (CTRL M, Z and B) and patients PT1, PT2 and PT3 were cultured in Dulbecco’s modified Eagle’s medium (DMEM) with high glucose (Euro Clone, ECB7501L), supplemented with 10% fetal bovine serum (FBS, Gibco, 100-18B-1MG), 1% penicillin/streptomycin (Gibco, ECB3001D), 1% L-glutamine (Gibco, ECB3000D), 10 μg/mL of Fibroblast grow factor (FGF, Peprotech, 100-18B-1 MG) and kept at 37°C in a 5% CO_2_ incubator. Pioglitazone (PGZ, Sigma-Aldrich, CDS021593-50MG) was dissolved in dimethyl sulfoxide (DMSO, Sigma-Aldrich, D2650-100ML) to a stock solution of 10 mM and added to the cell growth medium at the final concentration of 5 µM for 48 h. As untreated condition, cells were cultured with an equal volume of DMSO in the growth medium. The medium was exchanged every day with fresh medium. In some experiments, cells were treated with Insulin at the final concentration of 1 µM for 5 days and H_2_O_2_ at the final concentration of 800 µM for 48 h.

### 2.3 Mitochondrial membrane potential measurements and network evaluation

Measurement of alterations in the mitochondrial membrane potential (ΔΨm) was carried out by utilizing the JC1 staining kit (Sigma, CS0390) following manufacturer’s instructions. In cells with high ΔΨm, JC-1 molecules assemble into complexes referred to as J-aggregates, yielding a red to orange fluorescence (λ_em_ = 590 ± 17.5 nm), conversely, cells with low ΔΨm retain JC-1 in its monomeric form, resulting in solely green fluorescence (λ_em_ = 530 ± 15 nm).

Fibroblasts were cultured in glass-bottom dishes and incubated with JC1 in a 37°C, 5%CO_2_ incubator for 25 minutes, then washed twice with fresh PBS and incubated with phenol red-free basal medium for live imaging data acquisition.

### 2.4 Mitochondrial morphology

Mitochondrial morphology was analysed on TMRM-stained cells by using ImageJ software. Fibroblasts were cultured in glass-bottom dishes using fibroblast growth medium. Subsequently, the cells were washed with PBS and incubated at 37°C for 30 min in medium supplemented with a 20 nM TMRM (Invitrogen) and an additional 5 µM Hoechst dye (Invitrogen) to simultaneously stain the nuclei. Following the incubation period, the medium was removed, and the cells were washed with PBS. Images were captured using fluorescent confocal microscopy. All confocal images were obtained using a Leica SP8 microscope with identical acquisition parameters, specifically at an emission wavelength of 574 nm.

In brief, RGB images from fluorescent microscopy were converted into binary (black and white) images to define objects and background (segmentation), followed by filtering or preprocessing including noise reduction, background subtraction, contrast and feature enhancement filters. Particle Analysis was performed on selected region-of-interest (ROI), blanking the area outside of the selection, and thresholding. Morphological measures for each ROI were annotated (Merrill et al., 2017).

### 2.5 Quantitative RT-PCR

Total mRNA was isolated using an RNA isolation kit (Qiagen, Germany). Following the reverse transcription reaction with the Goscript Reverse Transcriptase kit (Promega, United States, A5001), a quantitative SYBR Green-based PCR reaction was performed using iTaq universal SYBR Green Supermix (Biorad, United States, 1725124) and the following conditions: 95°C for 2 min; 95°C for 15 s; 60°C for 1 min; 60°C for 31 s; 65°C for 5 s + 0.5°C/cycle, 39 cycles. The reaction was monitored with a CFX96 Real-Time PCR system (Biorad, United States). The expression level of each gene was normalized to the housekeeping gene ACTB encoding for b-actin. Fold changes in gene expression were calculated using the 2^−ΔΔCT^ method, based on biological reference samples and housekeeping gene for normalization. Primers are listed in Table 2.

**TABLE 2:** List of qPCR primers (5′–3′).

Gene	Forward	Reverse
*ACTB*	CCAACCGCGAGAAGATGA	CCAGAGGCGTACAGGGAT
*PITRM1*	CCA​TAC​CTG​TCA​CAG​AGT​TGG​AC	GGA​GTG​TGT​TCA​GGC​TGG​AGA​A
*MT-RNR1*	CCC​CAG​GGT​TGG​TCA​ATT​T	CTA​TTG​ACT​TGG​GTT​AAT​CGT​GTG

### 2.6 Western blot analysis

Proteins were extracted using an ice-cold 1X RIPA buffer supplemented with protease and phosphatase inhibitors from Roche (Switzerland). The extraction process involved centrifugation at 14,000 rpm and 4°C for 30 min, followed by three freeze-thaw cycles. The protein concentration in the resulting supernatant was determined using the Bradford protein assay kit from Biorad (CA, United States, 5000006). For gel electrophoresis, 30 μg of the protein lysate were loaded onto a polyacrylamide gel with a density ranging from 4% to 12%. The proteins were then transferred onto a nitrocellulose membrane. To block the blots, a solution of 5% milk powder or 5% BSA in TBS with 0.1% Tween-20 (TBST) was used. The membranes were incubated overnight at 4°C with primary antibodies diluted in either milk or BSA blocking solution. Subsequently, the membranes were incubated with corresponding HRP-conjugated secondary antibodies from Sigma-Aldrich (MO, United States) for 1 h at room temperature. Visualization of the proteins was achieved using either Clarity Western ECL Substrate (1705061) or Clarity Max Western ECL Substrate (1705062) from Biorad (CA, United States), and the images were captured with the Azure Biosystem Aerogene 300Q imaging system. Densitometric analysis was performed using ImageJ software: after background subtraction, the intensity of the signal of each band of the protein of interest was normalised to the signal of the corresponding loading control (GAPDH or VDAC1).

Primary antibodies included rabbit anti-PITRM1 (1:500, Atlas Antibodies #HPA006753, Sweden), rabbit anti-Frataxin (1:1,000, Abcam #ab175402, UK), rabbit anti-IDE (1:1,000, Abcam #ab228720,UK), rabbit anti-PMPCB (1:1,000, Abclonal #A4312, MA, United States), rabbit anti-GAPDH (1:1,000, Abcam #ab181602, UK) and rabbit anti-VDAC1 (1:1,000, Abcam #ab15895, UK); Rabbit Polyclonal PPARgamma/NR1C3 (1:100, Bio-techne #NB120-19481, MI, United States), Total OXPHOS Human WB antibody cocktail (1:500, Abcam, #ab110411), mouse anti-TFAM (1:1000, ThermoFisher MA5-16148). Secondary antibodies include ECL anti-mouse IgG peroxidase (Sigma-Aldrich, GENA931) and ECL anti-rabbit IgG peroxidase (Sigma-Aldrich, GENA9340).

### 2.7 DNA extraction and mtDNA copy number quantification

To quantify mtDNA, we collected cell pellets and extracted total DNA, using QIAamp DNA Micro Kit from Qiagen (#56304). Real-time quantitative PCR (qPCR) was performed by using SsoAdvanced universal Probes Kit (BioRad, #1725281), with primers and probe for the 12S subunit of mitochondrial rRNA (MT-RNR1) and TaqMan RNAse P control reagent Kit (Applied Biosystem, #4316844) as nuclear reference (Nasca et al., 2022). We performed three independent qPCR analysis for each cell line and for each condition. Primers sequences are listed in Table 2.

### 2.8 TC- FlAsH TFAM-Kate vector construction

To detect the accumulation of MTS through cell imaging we designed a dedicated fluorescent indicator based on biarsenical-binding tetracysteine motifs (TC). It has been shown that the (cell permeable) biarsenical reagents FlAsH-EDT2 can bind TC motifs. Upon binding, a thiol-arsenic ligand exchange reaction converts these nonfluorescent reagents into fluorescent, protein-bound, complexes (Griffin et al., 1998; Jakobs, 2006). We introduced a TC motif into the targeting sequence of the mitochondrial matrix protein TFAM, fused to the monomeric far-red fluorescent protein mKATE (Shcherbo et al., 2007; 2009). To design the probe, we first analysed the primary structure of TFAM by means of bioinformatic approaches to define the length of the MTS and predict the cleavage site of the mitochondrial matrix peptidase. According to MitoFates (Fukasawa et al., 2015) TPpred 2.0 (Savojardo et al., 2014) and TargetP 1.1 (Nielsen et al., 1997; Emanuelsson et al., 2000), the latter resides at amino acid 42, yielding the following MTS: MAFLRSMWGVLSALGRSGAELCTGCGSRLRSPFSFVYLPRWF. Implementation of the TC motif within this sequence was not random, as its insertion should not affect the properties of the subcellular localization sequence (e.g., efficiency of mitochondrial targeting) and of the cleavage site of the processing peptidase. We chose the smallest TC sequence (CCPGCC) sufficient for efficient FlasH binding as shown in (Griffin et al., 1998; Jakobs, 2006). We inserted it at position 21 after considering that the FlasH binding could take advantage not only of the TC motif that we inserted without changing the overall length of the MTS, but also of its flanking amino acids (Martin et al., 2005; Luedtke et al., 2007). We then confirmed though bioinformatic analyses that the new TFAM-MTS (which reads as follow: MAFLRSMWGVLSALGRSGAECCPGCCLRSPFSFVYLPRWF) was still able to drive localization within mitochondria (YLOC (Briesemeister et al., 2010a; Briesemeister et al., 2010b), iPSORT (Bannai et al., 2002), MitoFates, Target P 1.1, TPpred 2.0; scored respectively 68%, yes, 97%, 98%, 99% probabilities of mitochondrial localization/presence of peptidase cleavage site at position 42). A custom sequence including human TFAM (NCBI Reference Sequence: NP_003192.1), modified in its MTS as reported above and fused at its C-terminus with mKATE was inserted into the pUC57-Amp plasmid (gene synthesis from GENEWIZ, GENEWIZ South Plainfield, NJ). The fragment encoding for the TFAM-Kate was PCR amplified by using the forward (GGA​GGA​TCC​ATG​GCC​TTC​CTG​AG) and reverse (CCT​CTC​GAG​TCA​TCA​CCT​GTG​GC) primers, digested with BamHI and XhoI and cloned into the BamHI-XhoI sites of the pcDNA™5/FRT vector (Supplementary Figure S1). All restriction and modification enzymes were from Fermentas (St. Leon-Rot, Germany) unless otherwise stated.

### 2.9 In-cell biarsenical dye labelling

To label TC-tagged TFAM-Kate overexpressed in fibroblasts, the TC-FlAsH II In-Cell Tetracysteine Tag Detection Kit from Molecular Probes (Thermo Fisher Scientific) was utilized. The transfected CTRL B and PT3 cells were cultured in 35 mm glass-bottom dishes. For monitoring the effects over time, in-cell FlAsH labeling was conducted 48 h post-transfection. The cells were washed twice with reduced serum Opti-MEM without phenol red (Life Technologies) and then incubated with FlAsH at a concentration of 1 μM in Opti-MEM. This incubation took place at 37°C for 30 min, while being protected from light. After 30 min, the cells were washed twice with 1X BAL (2,3-dimercaptopropanol) buffer in Opti-MEM, again at 37°C and protected from light, for 1 h. Cells were stained with Hoechst (1 μg/mL) in Opti-MEM for 5 min. Finally, the cells were washed with Opti-MEM and prepared for live-cell imaging. The samples were visualized using an inverted laser scanning microscope (Axio Observer.Z1). Fiji software was employed for image analysis. Flash fluorescence images were segmented by automated color thresholding, enabling the identification of fluorescent positive particles within the cell cytoplasm. The average grey value of each particle was measured using the “analyze particles” command. Finally, the mean particle intensity per cell was calculated and used for statistical analysis.

### 2.10 Statistical analysis

All results are expressed as mean ± standard deviation (SD). Graph Pad Prism, version 7.03 software was used to generate the graphs and to perform statistical analyses, which are indicated in the corresponding figure legend.

## 3 Results

### 3.1 Patients’ PITRM1 variants cause protein instability and mitochondrial dysfunction

To evaluate the pathogenic effect of the PITRM1^R183Q^, PITRM1^V747Gfs*31^ and PITRM1^T931M^ variants (all together referred to as PITRM^MUT^), we first modelled the 3D structures of wild-type (WT) and mutated versions of PITRM1 using Pymol software.

**FIGURE 1:**
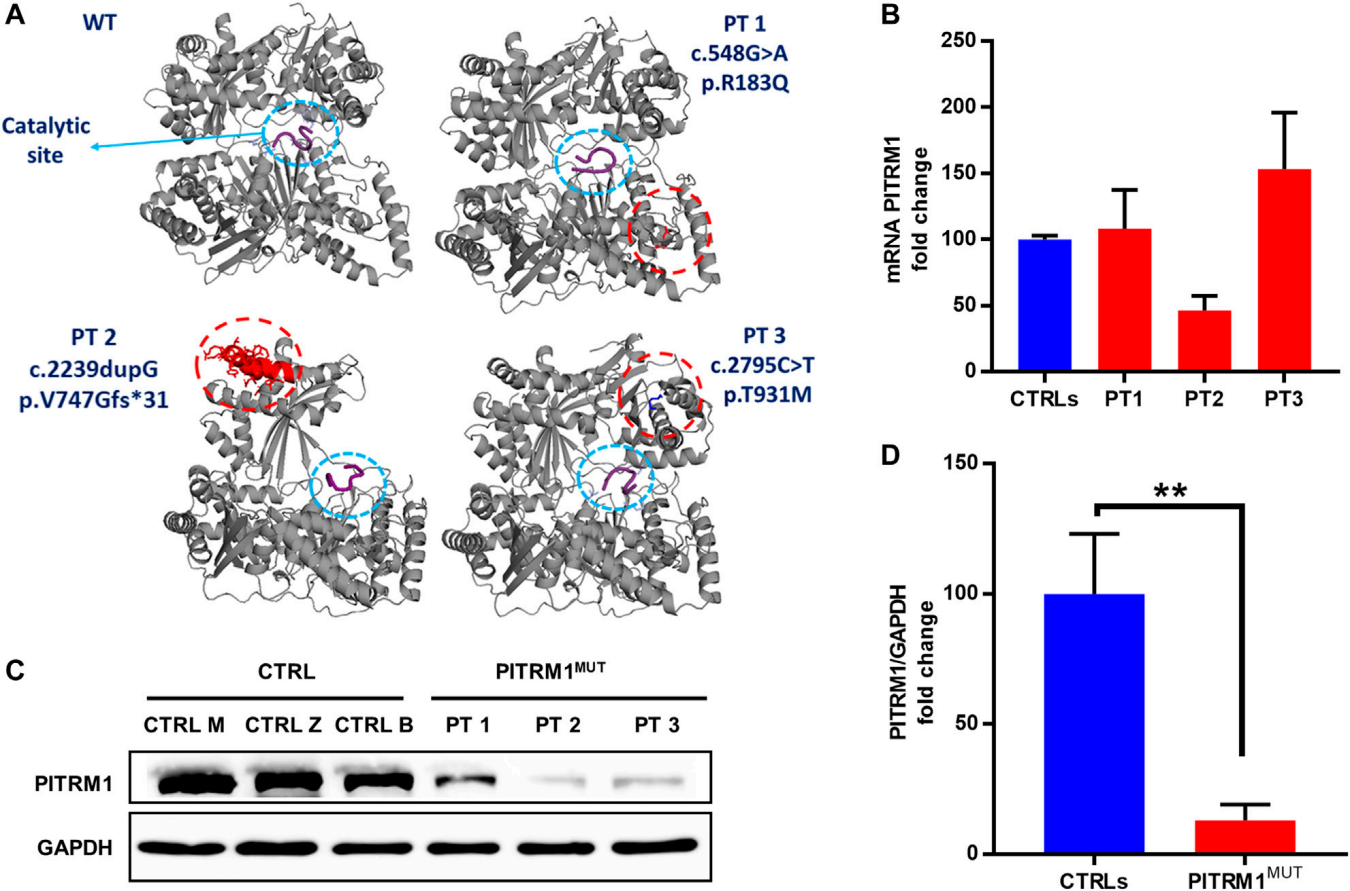
*PITRM1* pathogenic variants c.548G>A, c.2239dupG and c.2795C>T impact protein stability. **(A)** *In silico* reconstruction of PITRM1 structure for wild-type (WT) and mutated PITRM1 amino acid sequences. Patients’ variants are relatively distant from the catalytic site (blue circle) docked with β-amyloid peptide (purple). **(B)** *PITRM1* mRNA expression measured by qPCR did not show significant changes in PT1, PT2, and PT3 vs. CTRLs cells. **(C)** Western blot analysis of proteins separated by SDS–PAGE showed a marked reduction of PITRM1 amount in all fibroblasts suggesting protein instability. Quantifications of Western blot are shown in **(D)**. All data are expressed as fold change compared to controls; statistical analyses were performed with 1-way Anova followed by Tukey multiple comparisons test **(B)**, or with unpaired *t*-test **(D)**; **p < 0.005.

The *in silico* docking analysis of mutated protein *versus* Aβ peptide (a PITRM1 substrate) predicts that all three PITRM1 variants do not affect the catalytic site, suggesting that the PITRM1 enzymatic activity is preserved (Figure 1A). The *PITRM1* transcript levels were similar to control in PITRM1^R183Q^ (PT1) and PITRM1^T931M^ (PT3), but slightly reduced in PITRM1^V747Gfs*31^ (PT2) fibroblasts, although this difference was not statistically significant (fold changes: CTRLs: 100 ± 2.8; PT3: 46.3 ± 11, *p* > 0.05, Figure 1B). However, Western blot analysis of proteins separated by SDS–PAGE showed a marked reduction of PITRM1 amount in all patients (fold changes: CTRLs: 100 ± 23; PITRM1^MUT^: 12.9 ± 6.2, *p* < 0.005, Figures 1C, D), suggesting that all the three variants affect protein stability.

### 3.2 Presequence processing is impaired in PITRM1-mutant fibroblasts

Previous works done in yeast (Mossmann et al., 2014) and in human *PITRM1* KO cell lines (Kücükköse et al., 2021) showed that PITRM1 deficiency leads to the build-up of non-degraded MTSs that accumulate within the mitochondrial matrix, causing dissipation of the ΔΨm and impairing the processing of presequence proteins by the peptidase MPP.

**FIGURE 2:**
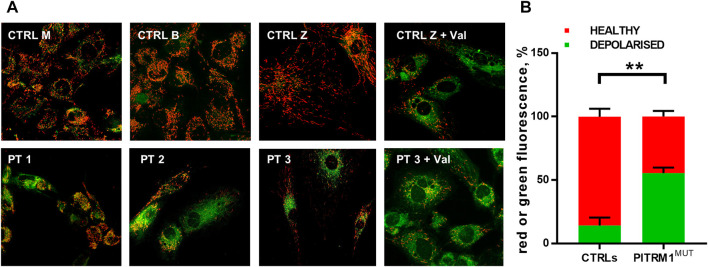
Mitochondrial membrane potential is reduced in PITRM1-mutated fibroblasts. **(A)** Healthy control cells stained with JC1 presented mainly red fluorescent aggregates indicating the preservation of the mitochondrial membrane potential. Fibroblasts derived from PITRM1-mutated patients presented a diffuse green fluorescence confirming the presence of defective mitochondrial membrane potential. Magnification 20x. Quantification of red or green fluorescence is shown in **(B)**. Statistical analysis was performed with 2-way Anova followed by Sidak’s multiple comparisons test; **p < 0.005.

To verify whether this proposed pathogenic mechanism is present also in the mutant patients, we evaluated ΔΨm by JC1 staining in PT 1, PT 2, and PT 3 fibroblasts (Figure 2A). The mitochondrial membrane potential was significantly compromised in PITRM1^MUT^ cell lines, which displayed a higher proportion of depolarised organelles compared to controls (depolarised/healthy mitochondria: CTRLs: 13.7%/85.3% ± 6.03%; PITRM^MUT^: 55.3%/44.7% ± 4.1%, *p* < 0.005, Figure 2B). This result confirms previous observations described in PITRM1^R183Q^ (Brunetti et al., 2016) and suggests that mitochondrial depolarisation is a common hallmark for *PITRM1* pathogenic variants.

**FIGURE 3:**
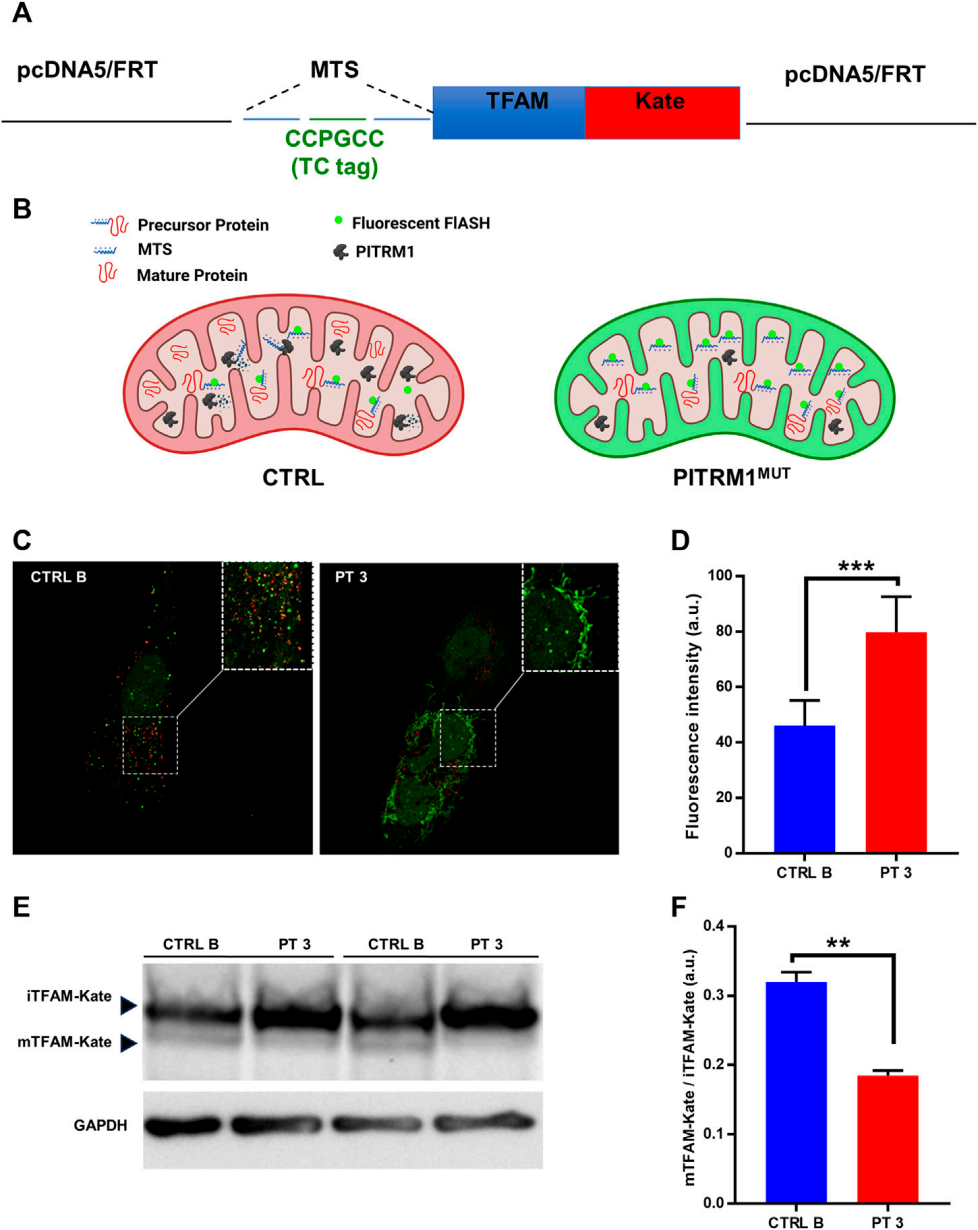
Presequence processing is compromised in PITRM1-mutated fibroblasts. **(A)** Schematic representation of the TC-FlAsH-TFAM-Kate reporter vector. **(B)** Schematic drawing showing the experimental rationale: degradation of MTS should impede detection of the FlAsH signal, whereas its accumulation should result in enhanced FlAsH-TC fluorescence. **(C)** Representative images showing that fibroblasts of PITRM1-mutated patient P3 accumulate undegraded MTS (green fluorescence) compared to CTRL B; magnification 40×; quantification is shown in **(D)**. **(E)** Representative Western blot analysis of TFAM-Kate processing; PITRM1-mutated fibroblasts show reduced levels of TFAM-Kate mature protein compared to CTRL B. GAPDH was used as a loading control. Quantification is shown in **(F)**. Statistical analysis was performed with unpaired *t*-test; **p < 0.005; ***p < 0.001.

MTSs can bind to the mitochondrial membrane and disrupt the electrochemical gradient of the mitochondria (Ståhl et al., 2002); therefore, to assess whether MTSs scavenging is compromised when PITRM1 is dysfunctional, we transfected CTRL B and PITRM1^T931M^ mutant fibroblasts (PT3) with the TC-FlAsH-TFAM-Kate reporter vector (Figure 3A). This probe holds a TC motif into the MTS of the mitochondrial matrix protein TFAM, fused to the monomeric far-red fluorescent protein mKATE (Figure 3A). Degradation of MTS should impede the detection of the FlAsH signal, whereas its accumulation should result in enhanced FlAsH-TC fluorescence (Figure 3B).

To determine whether FlAsH labelling was able to identify MTS-TC, we compared live-cell images after the addition of the FlAsH reagent to MTS-TC-TFAM-Kate expressing cells 48 h post-transfection (Figure 3C).

We measured the mean fluorescence intensity of Flash-TC and found that the intensity of the FlAsH-TC (green) signal was higher in the PITRM1^T931M^ mutant cell line compared to CTRL B (fluorescence intensity (arbitrary unit, a.u.): CTRL B: 46.15 ± 9.02; PT3: 79.82 ± 12.79, *p* < 0.001, Figure 3D).

To assess whether MPP presequence processing is compromised when PITRM1 amount is reduced, we analyzed by SDS-PAGE the levels of the exogenous presequence containing TFAM-Kate precursor, using an anti-TFAM antibody (Figure 3E). While the TFAM-Kate was processed by MPP in the control cells leading to the detection of cleaved form mTFAM, the amount of mature protein was strongly reduced in PITRM1^T931M^ mutant cells (CTRL B: mTFAM/iTFAM: 0.32 ± 0.01; PT3: 0.18 ± 0.007, *p* < 0.01, Figure 3F).

Altogether, these findings indicate that the proposed pathomechanism described in PITRM1-deleted yeast or in *PITRM1* KO cell models is also present in PITRM1^MUT^ fibroblasts.

### 3.3 Frataxin maturation is dysregulated in PITRM1-mutant fibroblasts

**FIGURE 4:**
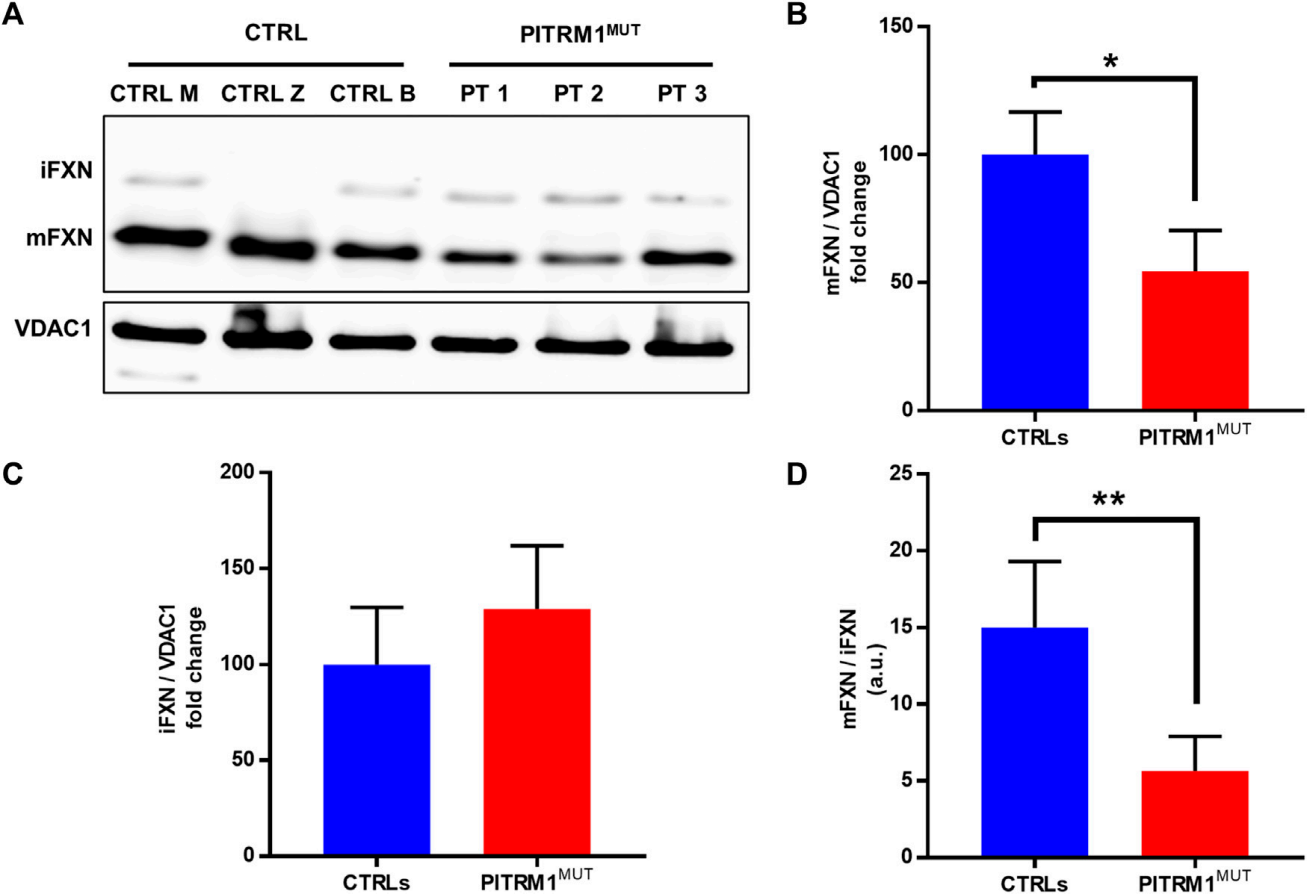
Frataxin processing is significantly reduced in PITRM1^MUT^ fibroblasts. **(A)** Respresentative Western blot analysis of Intermediated Frataxin (iFXN) and mature Frataxin (mFXN) in controls and PITRM1^MUT^cell lines. Quantification of **(B)** total mFXN, **(C)** total iFXN and **(D)** (mFXN/iFNX) are shown. VDAC1 was used as loading controls. In **(C)** and **(D)** data are expressed as fold change compared to controls; statistical analyses were performed with unpaired *t*-test; *p < 0.05, **p < 0.005.

Since mitochondrial peptidases are also involved in the maturation of the human Frataxin precursor, we examined Frataxin protein species, *i.e*., mature and immature forms (mFXN and iFXN, respectively), by immunoblotting in control *versus* patients fibroblasts (Figure 4A). *PITRM1*-mutant cells showed a significantly reduced amount of mature Frataxin (mFXN) compared to controls (fold changes: CTRLs: 100 ± 16.7; PITRM1^MUT^: 54.39 ± 16.05, *p* < 0.05, Figure 4B), as well as a non-statistically significant increase of iFXN form (fold changes: CTRLs: 100 ± 29.7; PITRM1^MUT^: 129 ± 33, *p* > 0.05, Figure 4C) which ultimately impaired the mFXN/iFXN ratio (CTRLs: 15.01 ± 4.29; PITRM1^MUT^: 5.66 ± 2.24, *p* < 0.01, Figure 4D), indicating a decreased function of MPP and defects of mitochondrial presequence processing.

To understand whether targeting PITRM1 enzymatic activity by external stressors may reduce Frataxin processing and mitochondrial membrane potential, we treated the PITRM1^R183Q^ cells with H_2_O_2_, which is known to reduce PITRM1 activity (Chen et al., 2014). Furthermore, since IDE could compensate for the PITRM1 deficiency by degrading MTSs as reported in yeast (Mossmann et al., 2014), we induced a pharmacological inhibition of this activity by “engulfing” IDE’s enzymatic activity with Insulin supplementation, as IDE has a higher affinity to Insulin than to MTS.

**FIGURE 5:**
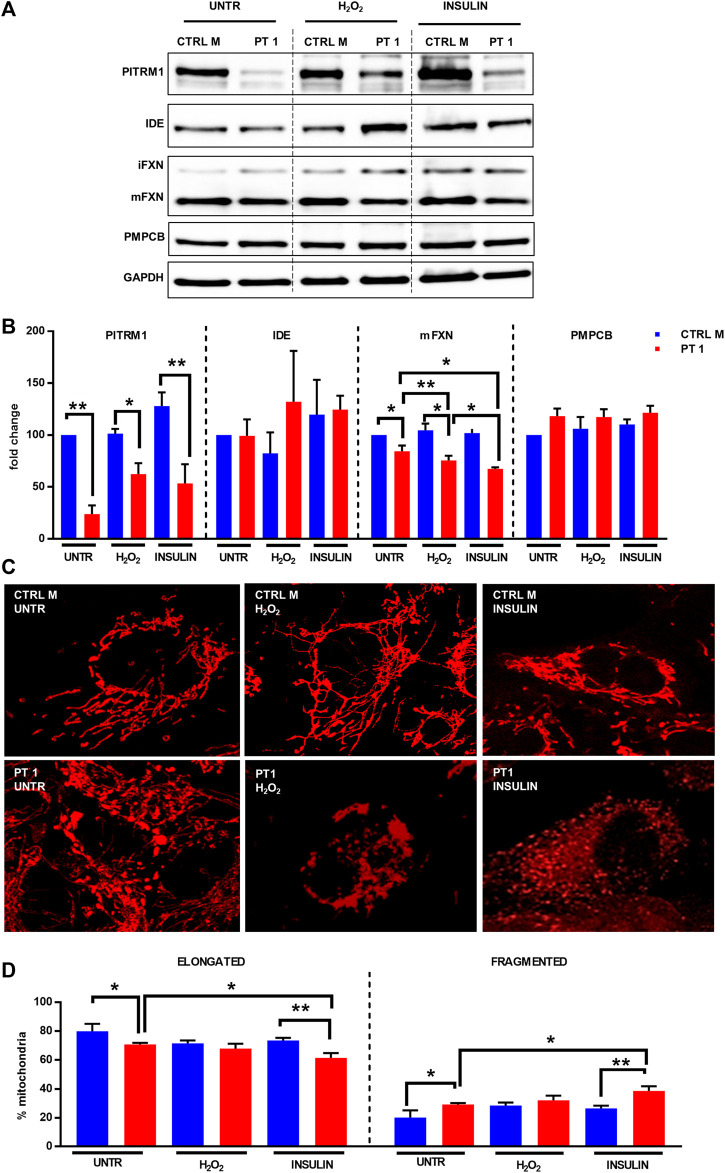
H_2_O_2_ and Insulin exacerbate defective mitochondrial proteostasis in PITRM1^MUT^ fibroblasts. **(A)** Representative western blot analysis of PITRM1, IDE, PMPCB, and Frataxin (FXN) in fibroblasts derived from a control (CTRL M) and patient 1 (PT1) exposed to H_2_O_2_ and Insulin for 48 h. Densitometric evaluations expressed as fold change compared to untreated CTRL M are reported in **(B)**, GAPDH was used as loading control. **(C)** TMRM staining in CTRL M and PT1 exposed to H_2_O_2_ or Insulin; magnification 40×. **(D)** Quantitative analysis of mitochondrial morphology (elongated *versus* fragmented mitochondria) in each experimental setting shown in **(C)**. Colour codes as in **(B)**. Statistical analyses were performed, for each gene, with RM-1-way Anova followed by Fisher LSD test **(C)**, or with 2-way Anova followed by Sidak’s multiple comparisons test **(D)**; *p < 0.05, **p < 0.005.

As revealed by immunoblot, PITRM1 protein levels were significantly reduced in PITRM1^R183Q^ fibroblasts and were not further modulated by H_2_O_2_ or insulin treatments (Figure 5A, B); No obvious changes were detected in IDE protein levels in both the CTRL and PITRM1^MUT^ cells (Figures 5A, B). This notwithstanding, the amount of mFXN was significantly reduced by both treatments in PITRM1^R183Q^ but not in CTRL cells (Figure 5B). This suggests that exogenous stressors targeting either PITRM1 or IDE have more detrimental effects on PITRM1-deficient cell lines, resulting in a reduction of MPP activity. Moreover, the amount of PMPCB (an MPP subunit) did not change (Figures 5A, B), thus excluding that the observed processing defects were caused by reduced amount of protein, thus confirming previous results obtained by others on *PITRM1* ^*KO*^ HEK293T cells (Kücükköse et al., 2021).

To better evaluate the impact of the impaired proteostasis exacerbated by the exogenous stressor agents on the mitochondria, we examined the mitochondrial network architecture (Figure 5C). First of all, untreated fibroblasts from PT 1 displayed mild yet significant alteration of the mitochondrial network, with a higher percentage of fragmented, and a lower percentage of elongated mitochondria compared to the control cell line (CTRL B: elongated/fragmented: 79.95%/20.05% ± 5.09%; PT1: elongated/fragmented 70.83%/29.17% ± 1.04%, *p* < 0.05, Figure 5D). The mitochondrial network architecture of control fibroblasts was not affected by insulin or H_2_O_2_ treatment (Figures 5C, D); on the contrary, PITRM1^R183Q^ cells displayed an increased network fragmentation with exogenous stressors (Figure 5C) which was exacerbated specifically by Insulin treatment (PT1 untreated: elongated/fragmented: 70.83%/29.17% ± 1.04%, insulin-treated PT1: elongated/fragmented: 66.50%/38.50% ± 3.27%, *p* < 0.01, Figure 5D).

Altogheter, these results suggest that oxidative stress and increased insulin levels are detrimental for PITRM1^MUT^ patients. The same deleterious effects may be expected during aging when the level of PITRM1 is lower (Brunetti et al., 2020).

### 3.4 Pioglitazone treatment restores preprotein processing and mitochondrial health

**FIGURE 6:**
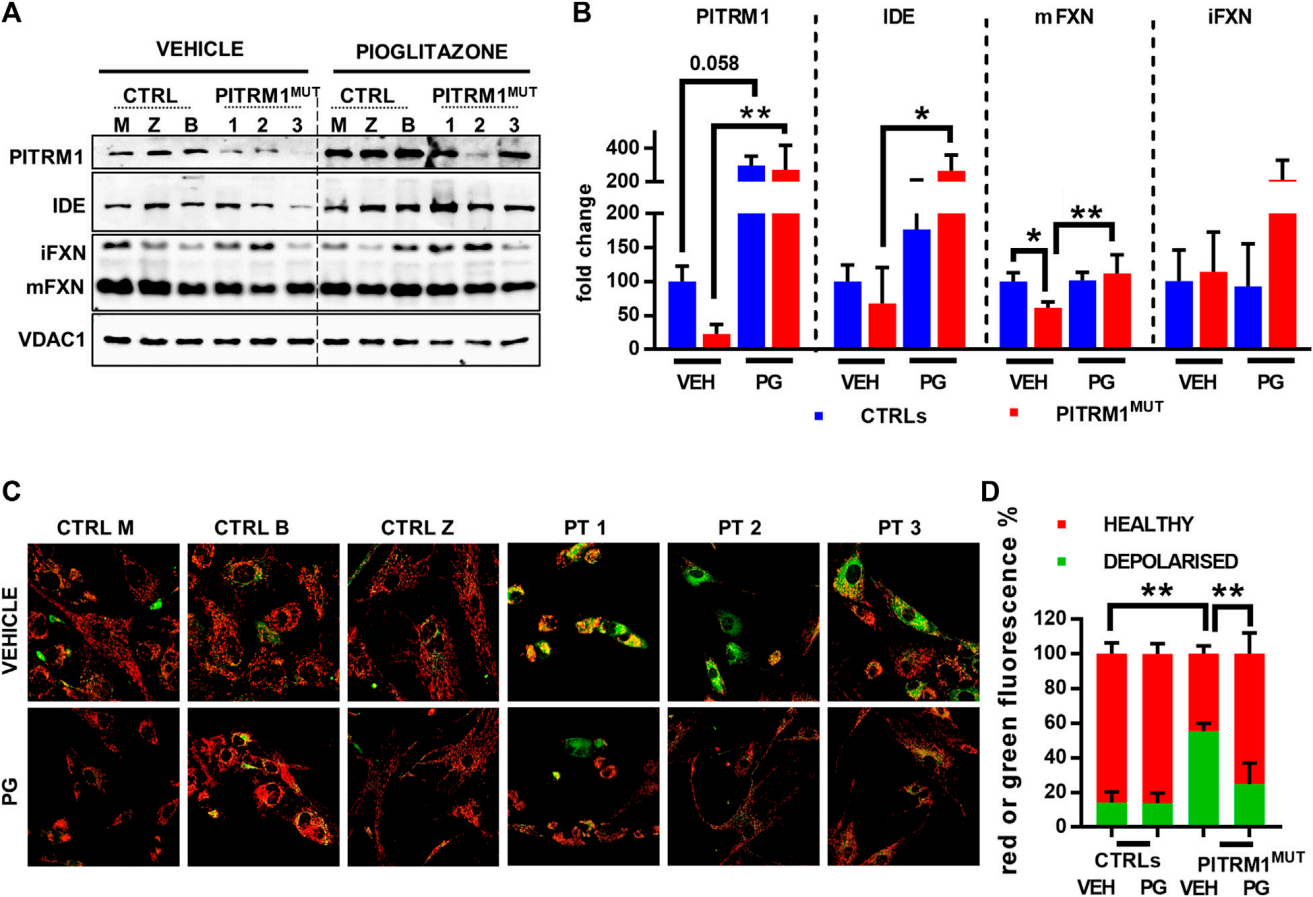
Pioglitazone treatment restores preprotein processing and mitochondrial membrane potential in PITRM1^MUT^ fibroblasts. **(A)** Representative Western blot analysis of pioglitazone (PG)-treated compared to vehicle (VEH)-treated fibroblasts. **(B)** Quantifications on the immunoblot signals normalised on VDAC1 signal and expressed as fold change compared to untreated CTRLs. PG upregulates IDE and PITRM1 levels that increase MTS degradation, restoring preprotein processing and Frataxin maturation. **(C)** Evaluation of mitochondrial membrane potential (JC1 fluorescence) in CTRLs and PITRM1^MUT^ fibroblasts exposed to vehicle or Pioglitazone for 48 h. Healthy control cells presented mainly red fluorescent aggregates indicating the preservation of the mitochondrial membrane potential. PITRM1^MUT^ fibroblasts presented with a diffuse green fluorescence indicating the presence of a defective mitochondrial membrane that is restored after pioglitazone treatment. Magnification: 20×. Statistical analysis was performed for each gene with 1-way Anova followed by Tukey’s multiple comparisons test **(B)**, or with 2-way Anova followed by Sidak’s multiple comparisons test **(D)**; *p < 0.05, **p < 0.005.

**FIGURE 7:**
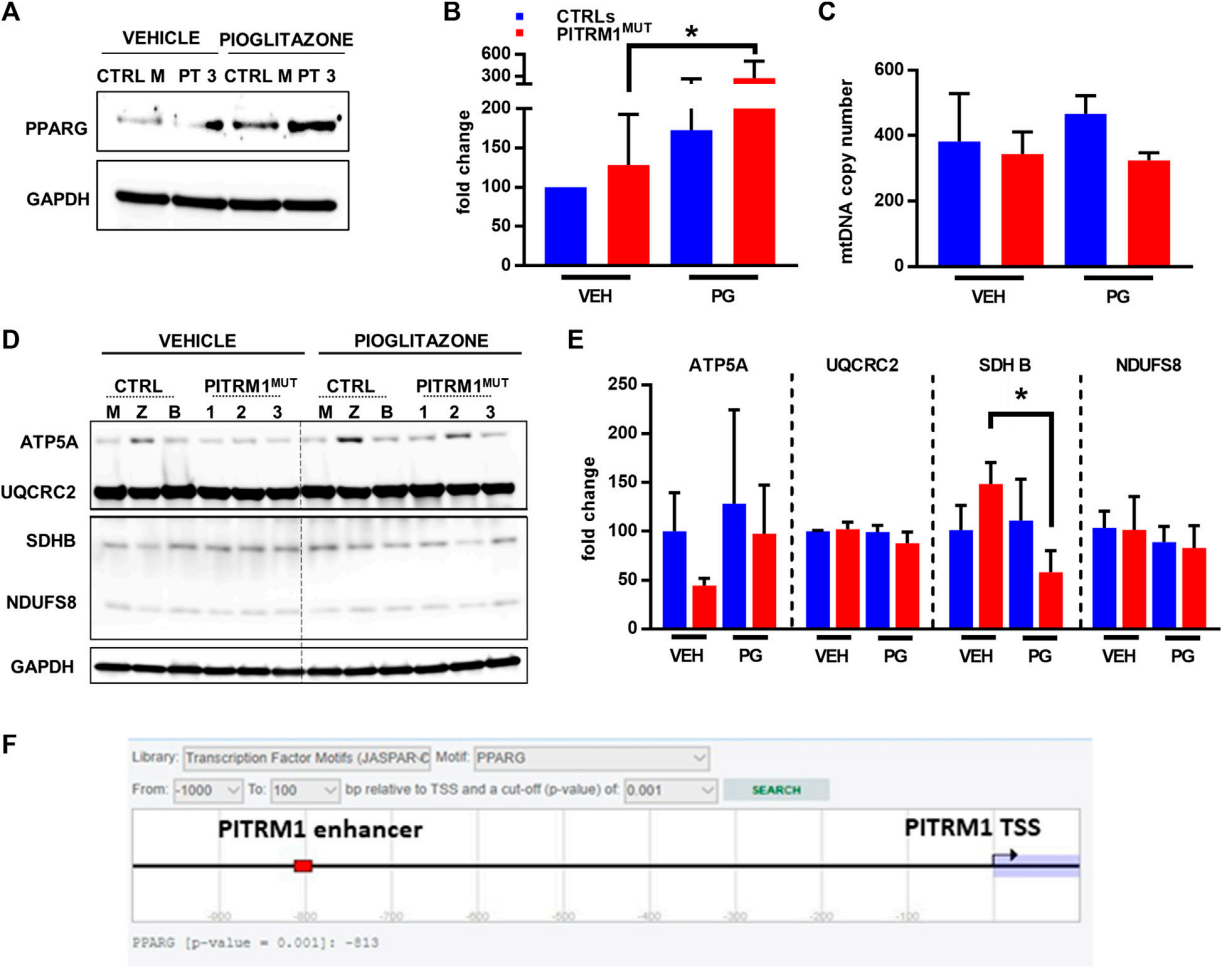
Pioglitazone enhances PITRM1 levels via PPARG. **(A)** Representative Western blot analysis of PPARG levels in CTRL and PITRM1^MUT^ fibroblasts; quantification is shown in **(B)**. **(C)** MtDNA quantification in control *versus* mutated cells w/ or w/o pharmacological treatment with Pioglitazone; colour codes as in **(B). (D)** Representative Western blot analysis of OXPHOS complex subunits in CTRLs and PITRM1^MUT^ fibroblasts, and quantification in **(E)**: no major differences were observed after treatment suggesting that Pioglitazone does not stimulate mitochondrial biogenesis in fibroblasts. Colour codes as in **(B)**. **(F)** Prediction of PPARG binding site with putative enhancer element close to PITRM1 TSS by EPD database (https://epd.epfl.ch/cgibin/get_doc?db=hgEpdNew&format=genome&entry=PITRM1_1). Statistical analysis was performed with 1-way Anova followed by Tukey’s multiple comparisons test; *p < 0.05.

Given that Ste23 (the homologue of human IDE in yeast), is required for efficient leader peptides degradation (Taskin et al., 2017), we assessed whether the pharmacological stimulation of IDE could act as a protective mechanism against MTS accumulation and defects of mitochondrial protein maturation. To this end, CTRLs and PITRM1^MUT^ fibroblasts were treated with Pioglitazone (PG), a PPARG agonist, with a reported effect on IDE upregulation. Indeed, Pioglitazone treatment (5 µM for 48 h) upregulated IDE levels improving Frataxin maturation specifically in PITRM1^MUT^ fibroblasts (fold changes PITRM1^MUT^: vehicle-treated: 67.97 ± 52.72; PG-treated: 267.9 ± 94.7, *p* < 0.05, Figures 6A, B). Surprisingly, we also detected a strong upregulation of PITRM1 levels, both in CTRLs (fold changes: vehicle-treated: 100.32 ± 22.16; PG-treated: 298.98 ± 54.96, *p* = 0.058) and in mutated cells (fold changes: vehicle-treated: 22.49 ± 14.06; PG-treated: 273.78 ± 145.33, *p* < 0.001, Figure 7B). In parallel, the maturation of Frataxin has also been restored in patients’ fibroblasts upon Pioglitazone treatment (fold changes: vehicle-treated: 61.36 ± 8,866; PG-treated: 112.17 ± 27.32, *p* < 0.005). To evaluate whether the upregulation of IDE and PITRM1 provided by Pioglitazone treatment was associated to an improvement of the mitochondrial membrane potential, we measured ΔΨm using JC1 staining. As shown in Figure 6C, control fibroblasts showed a preponderant emission at 590 nm wavelength (red fluorescence) in both conditions, indicating the preservation of the mitochondrial membrane potential (depolarised/healthy mitochondria: vehicle-treated: 14.1%/85.83% ± 6.29%; PG-treated: 13.88%/86.11% ± 5.87%, *p* > 0.05, Figure 6D). On the contrary, patients fibroblasts presented with a diffuse green fluorescence confirming the presence of a defective ΔΨm (Figures 6C, D), which interestingly, it was corrected by Pioglitazone treatment (depolarised/healthy mitochondria: vehicle-treated: 55.44%/44.55% ± 4.47%; PG-treated: 24.97%/75.02% ± 12.01%, *p* < 0.005, Figure 6D) and became indistinguishable from control cells (Figures 6C, D).

Thus, we examined the expression levels of PPARG protein in basal conditions (only vehicle, VEH) and after PG supplementation (Figure 7A); we found that PPARG was significantly upregulated in patients’ fibroblasts after PG treatment (fold changes: vehicle-treated: 128.3 ± 64.24; PG-treated: 279.6 ± 231.2 *p* < 0.05, Figure 7B).

Since Pioglitazone has been proposed to activate mitochondrial biogenesis (Corona and Duchen, 2016) we evaluated whether the increase in PITRM1 protein level observed after pioglitazone treatment was related to an increase in mitochondrial content. However, no significant differences in mitochondrial DNA copy number (Figure 7C) or in mitochondrial OXPHOS proteins were observed in VEH- *versus* PG-treated cells from both controls and patients (Figures 7D, E). Only SDHB (subunit of complex II) was significantly reduced in patients’ fibroblasts upon PG treatment (fold changes: vehicle-treated: 148.4 ± 21.93; PG-treated: 57.91 ± 22.34, *p* < 0.05, Figure 7E).

Overall, our results suggest that *PITRM1* gene is modulated by PPARG via a transcriptional mechanism. This hypothesis is supported by an *in silico* prediction revealing a significative binding score (*p* < 0.001) between PPARG and a nucleotide sequence (predicted as a *PITRM1* enhancer) located at −813bp upstream the *PITRM1* Transcription Starting Site (Figure 7F).

## Discussion

Several studies in yeast and human cellular models showed that PITRM1 deficiency is associated with the accumulation of MTSs in the mitochondrial matrix which impair the maturation of mitochondrial proteins by backlogging the function of MPP (Kücükköse et al., 2021).

We have recently described patients from three unrelated families carrying pathogenic variants in *PITRM1*, resulting in progressive spinocerebellar ataxia, obsessional behaviour, and cognitive decline (Brunetti et al., 2021).

Here, we confirmed the proposed pathogenic mechanism of PITRM1 deficiency in patients’ fibroblasts holding the three known *PITRM1* pathogenic variants. Common features found in all 3 cell lines are: dysfunctional mitochondrial membrane potential associated to impaired processing and maturation of imported proteins including Frataxin. Further, we provided evidence of MTSs accumulation in *PITRM1* mutant fibroblasts.

Understanding whether the dysregulated Frataxin maturation observed in *PITRM1* patients represents a reliable biomarker or may have a direct pathogenetic role in the onset of the ataxic phenotype is of primary importance and may be useful to understand part of the pathogenic mechanisms of Friedrich’s ataxia. Interestingly, impaired maturation of Frataxin was also reported in patients affected by mutations in the catalytic PMPCB subunit of MPP who present neurological regression and ataxia with basal ganglia lesions and cerebellar atrophy (Vögtle et al., 2018; Brunetti et al., 2021).

An important point emerging from our analysis is that the reduction of membrane potential, caused by the accumulation of MTSs, does not induce a reduction of nuclear proteins imported into the mitochondria. However, this result did not emerge clearly even in previous studies conducted on PITRM1 KO cells: in a recent study performed on HEK293T PITRM1 KO cells, a mildly reduced import capacity was observed (Kücükköse et al., 2021), however in PITRM1 KO neural progenitor cells and neurons an evident import defect was not detected (Pérez et al., 2021). If an import defect was not so evident in cell lines in which PITRM1 is completely absent (PITRM1 KO), it can be assumed that it is even less likely to observe this defect in fibroblasts from patients in which a minimal amount of functional PITRM1 is still present. This hypothesis is retrospectively confirmed by our previous study on PITRM1 heterozygous mice (Brunetti et al., 2016) in which this defect did not emerge. Further studies will be needed to evaluate the compensatory activity of other matrix proteases (i.e., IDE or Neurolysin) or the function of the mitochondrial stress response in keeping the mitochondrial protein homeostasis intact.

It has been previously demonstrated that impaired MTS processing and accumulation of immature precursor proteins caused by *CYM1* (homologue of *PITRM1*) deficiency, could be restored by overexpressing the mitochondrial isoform of Ste23 (homologue of human *IDE*) in yeast. In addition, recent reports have shown that the human *IDE* promoter is a direct target of the Peroxisome proliferator-activated receptor gamma (PPARG) (Du et al., 2009). The idea of exploiting pharmacological stimulation of IDE to compensate for PITRM1 deficiency, and improve preprotein processing, was verified on the patients’ cell lines using the PPARG agonist Pioglitazone, which upregulated IDE levels restoring mitochondrial membrane potential and improving Frataxin maturation. Surprisingly, we also detected an upregulation of PITRM1 levels.

Since we did not observe a parallel increase of other mitochondrial proteins or mtDNA copy number, we propose that the increased levels of PITRM1 did not depend on the increase of mitochondrial biogenesis but rather on direct action of PPARG on *PITRM1* gene expression.

*In silico* prediction revealed a significant binding score between PPARG and a nucleotide sequence predicted as a *PITRM1* enhancer. Of note, *PITRM1* and *IDE* are paralogous genes, both located on Chr10, which encode for two metallopeptidases with a similar molecular weight and function. So, it can be hypothesized that if *IDE* is a Pioglitazone-induced PPARG target, then *PITRM1* can be a pioglitazone target as well.

Pioglitazone (ATC code A10BG03) belongs to the family of thiazolidinediones (TZD), a group of heterocyclic drugs with a strong affinity for the PPAR receptor that are used to treat type 2 diabetes mellitus. Once active, the RXR receptor and PPAR bind to DNA, and this heterodimer interacts with PGC-1α and other transcriptional coactivators (Bottani et al., 2020). This marketed drug could be quickly repurposed to treat *PITRM1* patients. Usually, repurposing existing drugs for rare disease is a more rapid, and more successful approach than developing new orphan drugs. However, recently, a novel brain penetrant and orally bioavailable PPARG agonist named Leriglitazone (MIN-102) was successfully tested in cellular and animal models of Friedreich ataxia. In Frataxin-deficient dorsal root ganglia (DRG) neurons, the administration of leriglitazone led to an increase in Frataxin protein levels which was accompanied by a reduction in neurite degeneration and an improvement in cell survival. These effects resulted in an overall amelioration in mitochondrial functions and calcium homeostasis. Furthermore, leriglitazone demonstrated enhanced efficacy in treating central nervous system (CNS) diseases, as evidenced by its ability to improve motor function and restore mitochondrial function and biogenesis in animal models of Friedreich ataxia and adrenomyeloneuropathy (Rodríguez-Pascau et al., 2021).

Although further studies are required to fully elucidate the pathogenic mechanism triggered by PITRM1 and to test the efficacy of PPARG agonists in restoring preprotein processing *in vivo* our new data pave the way to develop a potential treatment for this rare and complex neurodegenerative disorder. Possibly, other common neurological conditions may benefit from this pharmacological approach.

## Data Availability

The raw data supporting the conclusion of this article will be made available by the authors, without undue reservation.
