# Supplementary figures and images for: PPAR-gamma agonist pioglitazone recovers mitochondrial quality control in fibroblasts from PITRM1-deficient patients
Source: Front Pharmacol. 2023 Jul 26;14:1220620. doi: 10.3389/fphar.2023.1220620 (PMC10415619; doi:10.3389/fphar.2023.1220620)

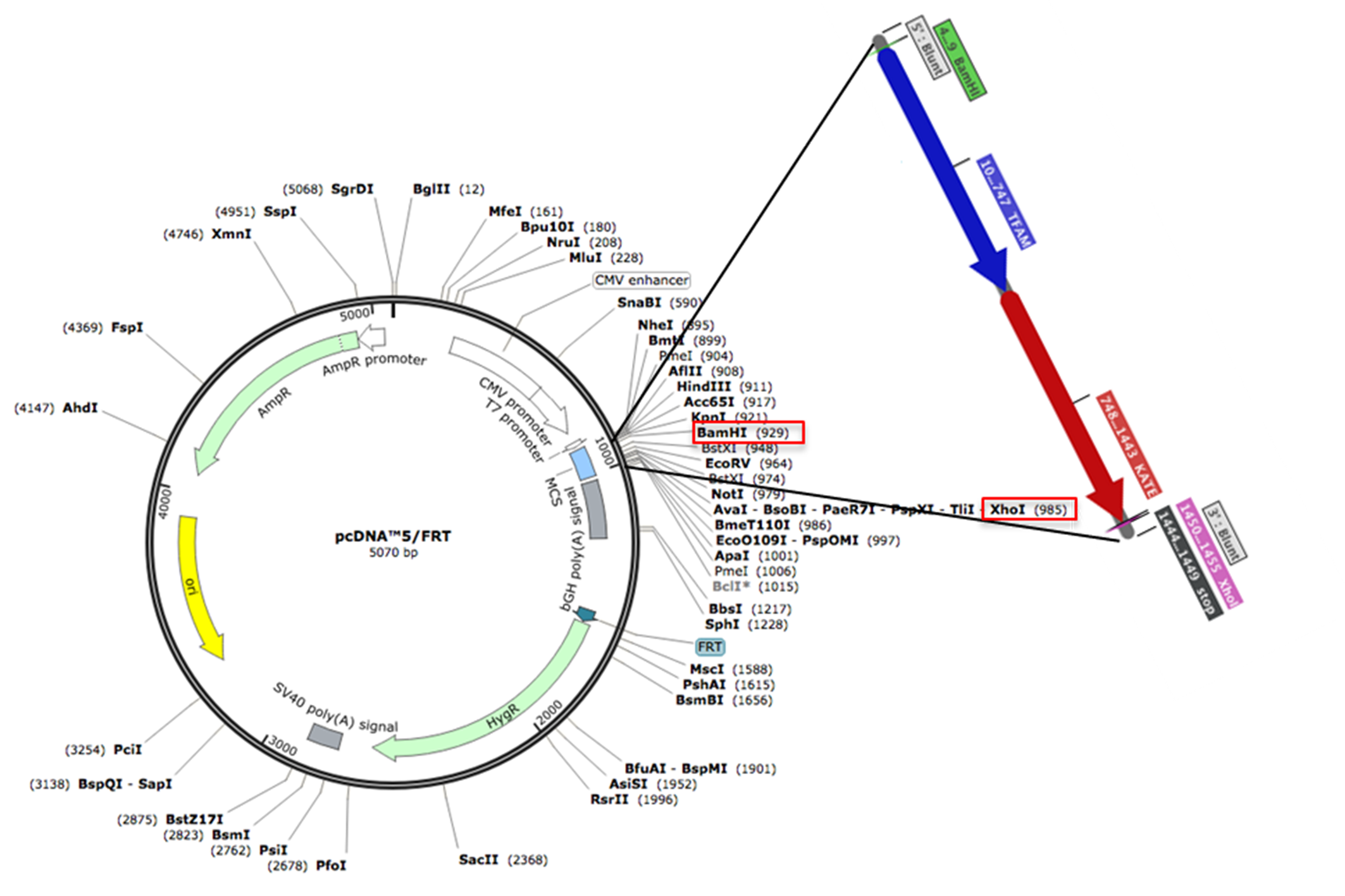

Supplement: Supplementary file 1 [file Image1.TIF]
